# An Unusual Left Upper Quadrant Mass: A Bronchopulmonary Foregut Malformation

**DOI:** 10.1155/2013/740292

**Published:** 2013-02-27

**Authors:** R. L. McDermott, D. O. Kavanagh, W. Bartosik, C. Quinn, P. R. O'Connell

**Affiliations:** ^1^Department of Colorectal Surgery, St. Vincent's University Hospital, Elm Park, Dublin 4, Ireland; ^2^Department of Cardiothoracic Surgery, St. Vincent's University Hospital, Elm Park, Dublin 4, Ireland; ^3^Department of Histopathology, St. Vincent's University Hospital, Elm Park, Dublin 4, Ireland; ^4^Department of Surgery and Surgical Specialties, St. Vincent's University Hospital, Elm Park, Dublin 4, Ireland

## Abstract

We report a case of a lady who presented with epigastric discomfort. Physical examination revealed a large left upper quadrant mass filling the left upper quadrant. Following extensive preoperative evaluation, she underwent resection of this 9 × 10 × 11 centimeter mass with *en bloc* excision of a portion of the left hemidiaphragm. She made an uneventful postoperative recovery. Histopathology revealed a bronchopulmonary foregut malformation with pulmonary sequestration. This developmental anomaly of the foregut typically occurs in the thoracic cavity; however, it can occur below the diaphragm. Herein we report a case and a detailed review of the embryology, clinical features, and management of these extremely rare clinical entities.

## 1. Introduction

Cysts and duplications are common anomalies of development. A bronchopulmonary sequestration is a rare congenital malformation of the lower respiratory tract that consists of a nonfunctioning mass of lung tissue that lacks normal communication with the tracheobronchial tree [1]. Depending on their site, sequestrations are classified as intralobar (when the lesion is located within a normal lobe and lacks its own visceral pleura) or extralobar (where the mass is located outside the normal lung and has its own visceral pleura). 

The term bronchopulmonary foregut malformation is used to broadly encompass a spectrum of embryological lesions that include bronchopulmonary sequestration, tracheal stenosis, bronchogenic cysts, congenital cystic adenomatoid malformations, bronchial atresia/stenosis, and congenital lobar emphysema. The relative incidence of bronchopulmonary sequestration within the spectrum of bronchopulmonary foregut malformations is 27% [2]. Approximately, 10%–15% of extralobar sequestrations are found within or below the diaphragm [3].

Retroperitoneal bronchogenic cysts despite their rarity occur with equal frequency among genders across a wide age range. The reported incidence of such extralobar, subdiaphragmatic malformations is 1 per 10000 [4] with the oldest case occurring in a 62-year-old man [5] and the youngest being a 3-month-old girl (diagnosed antenatally at 23 weeks of gestation) [6].

The majority of reported cases are incidental findings on imaging modalities and measure less than 5 cm in diameter. The commonest presenting symptom is epigastric or back pain similar to the current case, but these occurred in cysts greater than 7 cm. The largest reported case in the medical literature claims a maximum dimension of 10 cm [5, 7]. Our case surpasses this with maximum dimension of 11 cm. 

## 2. Case Report

A 48-year-old female presented to the emergency department with a two-month history of worsening epigastric discomfort. She had no associated symptoms. Clinical examination revealed a firm, tender mass in the left upper quadrant. It was nonpulsatile, dull to percussion, and moved inferiorly with respiration. It was not possible to palpate a superior edge while the lower margin was palpable. She had previously undergone a right ovarian cystectomy for a benign dermoid cyst. Initial haematological and biochemical indices were all investigated and within normal parameters.

Ultrasound revealed a large (9 cm × 10 cm × 11 cm), poorly vascularised mass, separate from the spleen, containing mixed solid and cystic elements. Internal septations were also apparent. 

**Figure 1:**
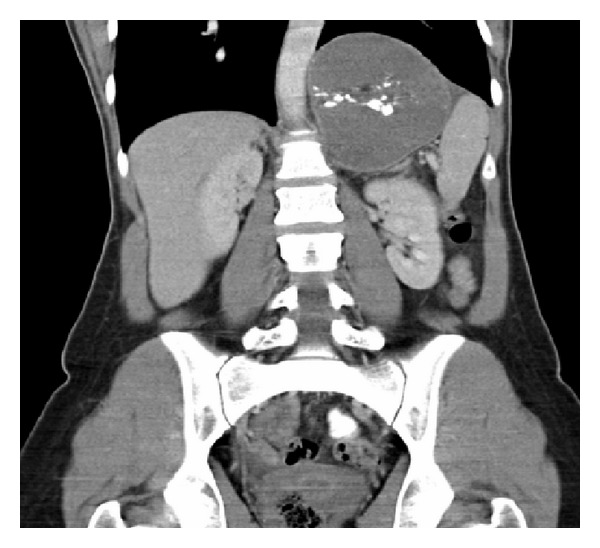
Computer tomography revealed a multiseptated cystic mass in the left upper quadrant with multiple foci of calcification within its walls that displaced the left hemidiaphragm superiorly.

CT imaging (Figure 1) demonstrated a multiseptated cystic mass with multiple foci of calcification within its walls. There were associated elevation of the left hemidiaphragm and displacement of the pancreatic tail and stomach anteriorly and the spleen laterally. 

**Figure 2:**
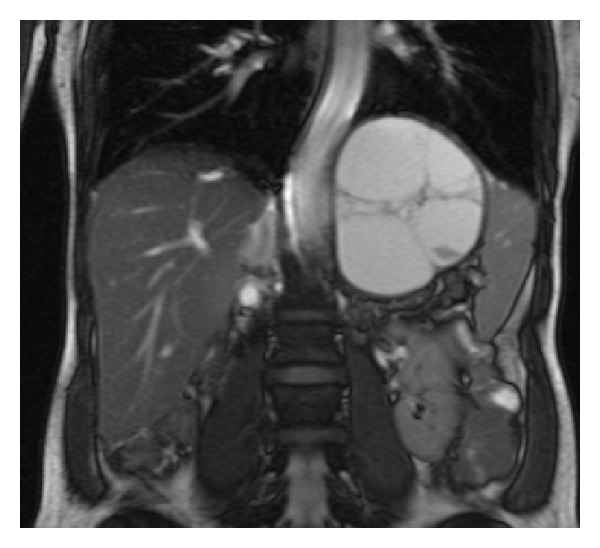
High signal on T2-weighted magnetic resonance imaging.

MR imaging (Figure 2) showed high signal on T2-weighted views and confirmed its separation from spleen, stomach, pancreas, and adrenal glands. There was no vascular invasion or associated lymphadenopathy. 

Since the mass was causing significant discomfort and of uncertain malignant potential, a decision was made to excise this mass. The patient was placed in a supine position. An upper midline incision facilitated adequate access to the left upper quadrant. The cystic mass was adherent to the membranous portion of the left hemidiaphragm. This was excised *en bloc* following aspiration of 700 millilitres of viscid fluid. An intercostal drainage tube was placed in the left hemithorax attached to an underwater seal and the diaphragmatic defect was closed with 2/0 polydioxanone. She made a favourable postoperative recovery and was discharged home on the 6th postoperative day. She remained well at outpatient followup.

**Figure 3:**
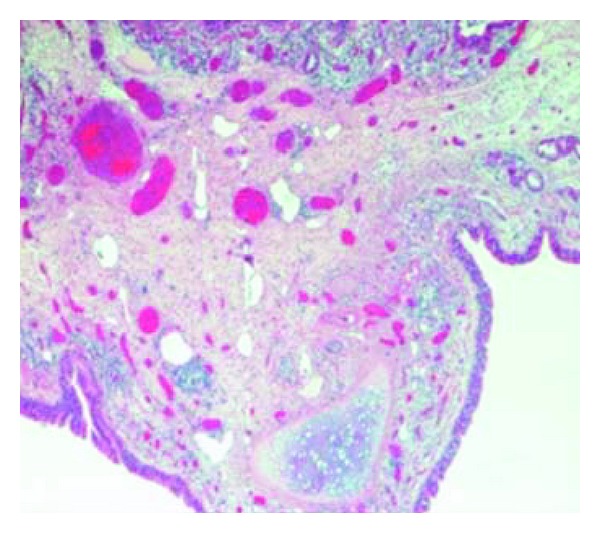
Haematoxylin and Eosin staining of the multiloculated structure showing it lined by ciliated respiratory-type epithelium with florid histiocytic infiltration and a granulomatous configuration.

Haematoxylin and Eosin analysis revealed that the multiloculated structure was lined by ciliated respiratory-type epithelium with a florid granulomatous reaction (Figure 3). Deposits of calcium and amorphous degenerative material were seen within the cystic spaces. Islands of cartilage and adipose were also noted between the cystic spaces. The final histological diagnosis was a benign bronchopulmonary foregut malformation.

## 3. Discussion

Gerle et al. in 1968 first used the term “bronchopulmonary foregut malformation” and described 13 cases of pulmonary sequestration communicating with the gastrointestinal tract [8]. However, it was not until 1976 that Heithoff et al. expanded the term to encompass a spectrum of pathological entities that were alluded to in the introduction [9].

It is generally accepted that foregut malformations arise from a supernumerary lung bud that arises caudally to the normal lung bud and migrates caudally along with the developing oesophagus. If the accessory lung bud arises before the development of the pleura, it becomes invested in the adjacent normal lung forming an intralobar sequestration. Conversely, if it develops later, after the formation of the pleura, it grows separate from the adjacent lung surrounded by its own pleura, becoming an extralobar sequestration. This process occurs during the 3rd–7th weeks of development [9]. The exact mechanism as to why such lesions lie in the retroperitoneum is unknown; however, Sumiyoshi et al. hypothesised that a portion of the tracheobronchial tree could be “pinched off” by the forming of pleuroperitoneal membranes (the future diaphragm) with resultant caudally migration through the pericardioperitoneal canal [4]. 

The differential diagnosis of a left upper quadrant mass is extensive and beyond the confines of this case report but the differential of a retroperitoneal cyst lined with pseudostratified, ciliated columnar epithelium includes cystic teratoma (only six reported cases) [5], bronchopulmonary sequestration, cysts of urothelial and Mullerian origin, and other foregut cysts [6]. Differentiating histological factors includes the following: teratomas have tissue from the three germinal layers; pulmonary sequestration has lung parenchyma within pleural investment; Mullerian or urothelial cysts have subepithelial cartilage and seromucous glands; well-developed smooth muscle layers suggest oesophageal cysts. 

Presentation is entirely variable and depends upon the type, size, and location. They are often detected on antenatal ultrasonography. Hydrops occasionally occurs, presumably as a result of vascular compression. In a series of 41 pregnancies whereby extralobar sequestration was detected antenatally [10], the lesion was detected during the second trimester on routine screening, the lesion resolved in 71.8% (28/39) of pregnancies, and hydrops was present in 9.7% (4/41). Postnatal presentation can be pneumonia, bleeding, pain, swelling, or incidental finding on imaging modalities.

The commonest sites of communication of these retroperitoneal lesions are in the left para-adrenal region with lower oesophageal and cardiooesophageal junctions (83%) [9]. Blood supply is variable with thoracic aorta, abdominal aorta, and pulmonary artery branches being the most frequent supply vessels [9].

Surgical resection is the standard treatment to alleviate symptoms and in case if concern exists regarding its malignant potential [11, 12]. Surgical approach to extralobar sequestrations involves identifying all feeding vessels and removing the lesion and its investing pleura. Arterial embolization has also been described in the management of these lesions [13, 14].

Owing to the paucity of the published literature on this topic, little is known about the outcome following resection. Patients with intrathoracic sequestrations did very well without significant complications. The majority morbidity was recurrent respiratory tract infections [15].

In conclusion, extrathoracic bronchopulmonary malformations are rare benign pathological entities. These can reach a substantial size prior to presenting with nonspecific symptoms. Despite detailed preoperative imaging, surgical extirpation is essential to alleviate symptomatology and elucidate its malignant potential.
